# “An ethnographic exploration of factors that drive policing of street-based female sex workers in a U.S. setting - identifying opportunities for intervention”

**DOI:** 10.1186/s12914-020-00232-0

**Published:** 2020-05-14

**Authors:** Katherine H. A. Footer, Bradley E. Silberzahn, Sahnah Lim, Steven Huettner, Victor A. Kumar, Derek Loeffler, Sarah M. Peitzmeier, Susan G. Sherman

**Affiliations:** 1 Johns Hopkins Bloomberg School of Public Health, Department of Health, Behavior and Society, 624 N Broadway, Baltimore, MD 21205 USA; 2 New York University School of Medicine, Department of Population Health and Medicine, 550 First Avenue, New York, NY 10016 USA; 3 Johns Hopkins School of Medicine, Department of Pediatrics, 5200 Eastern Ave, Baltimore, MD 21224 USA; 4 Johns Hopkins Krieger School of Arts and Sciences, Department of Anthropology, 466 Mergenthaler Hall, 3400 N. Charles Street, Baltimore, MD 21218 USA; 5 Baltimore City Police Department, Northwestern District, 5271 Reistertown Road, Baltimore, MD 21215 USA; 6 Johns Hopkins Bloomberg School of Publissc Health, Department of Population, Family, and Reproductive Health, Baltimore, MD USA

**Keywords:** Sex work, Cisgender female, Policing, Structural interventions, Health, Human rights

## Abstract

**Background:**

Building on a broader sociological discourse around policing approaches towards vulnerable populations, increasing public health and human rights evidence points to policing practices as a key health determinant, particularly among street-based sex workers. Despite the importance of policing as a structural health determinant, few studies have sought to understand the factors that underlie and shape harmful policing practices towards sex workers. This study therefore aimed to explore the drivers for policing attitudes and practices towards street-based cisgender female sex workers.

**Methods:**

Drawing on ethnographic methods, 280 h of observations with police patrol and 10 stakeholder interviews with senior police leadership in Baltimore City, USA were carried out to better understand the drivers for policing strategies towards cisgender female sex workers. Analysis was data- and theory-driven, drawing on the concepts of police culture and complementary criminological and sociological literature that aided exploration of the influence of the ecological and structural environment on policing practices.

**Results:**

Ecological factors at the structural (e.g., criminalization), organizational (e.g., violent crime control), community and individual level (e.g., stigmatizing attitudes) emerged as key to shaping individual police practices and attitudes towards cisgender female sex workers in this setting. Findings indicate senior police support for increased alignment with public health and human rights goals. However, the study highlights that interventions need to move beyond individual officer training and address the broader structural and organizational setting in which harmful police practices towards sex work operate.

**Conclusions:**

A more in-depth understanding of the circumstances that drive law enforcement approaches to street-based sex work is critical to the collaborative design of interventions with police in different settings. In considering public health-police partnerships to address the rights and health of sex worker populations in the U.S. and elsewhere, this study supports existing calls for decriminalization of sex work, supported by institutional and policy reforms, neighborhood-level dialogues that shift the cultural landscape around sex work within both the police and larger community, and innovative individual-level police trainings.

## Background

The criminal justice system has been identified as a prominent apparatus for reinforcing stigmatization amongst vulnerable groups [1]. Goffman wrote that stigma removes a persons’ ‘social acceptance’ leaving them with a spoiled identity’ [2]. Stigmatization includes forms of labelling, stereotyping and discrimination [3] that lead to both individual and social exclusion, which has in turn been linked to a range of negative physical and mental health outcomes for vulnerable populations, including sex workers [4, 5]. Police interactions are an omnipresent feature in the lives of many marginalized communities, shaping their everyday existence [6]. Extensive criminological and sociological literature has explored the role of policing in the social control of urban spaces and the communities within them (e.g., homeless persons, black youth, people who use drugs) [7–10]. These works have included an examination of how policing drives socio-economic exclusion and ‘system avoidance’ whereby marginalized groups fail to access healthcare and other forms of social support that make up fundamental social rights [11]. Street-based cisgender female sex workers (FSW) represent one such important marginalized group, whose working conditions (e.g., criminalization of sex work, community stigma, client violence, frequent drug use) [12] place them in frequent contact with the police. Criminalization and police enforcement of sex work has increasingly been associated with negative health-related outcomes and human rights abuses among FSW including, increased risk of experiencing different forms of violence and sexually transmitted diseases [13].

FSW have been identified as a population who face a complex constellation of structural vulnerabilities (e.g., poverty, low health care access, exposure to violence, drug use) [14]. Within this broader portrait of social and material adversity, research has begun to map the pathways by which policing of FSW influences individual (e.g., condomless sex) and interpersonal (e.g., client violence) HIV related risk [15, 16]. Laws relating to sex work furnish police with considerable discretion, which in turn has been shown to lead to exploitation in many contexts [17]. Across different global settings research has documented human rights abuses by police towards FSW, including harassment, sexual and physical violence, and coerced sex under the threat of arrest [18]. These types of violations impact women’s safety (undermining FSWs’ ability to seek police protection) and HIV risk (police-perpetrated sexual violence is often unprotected and associated with STI/HIV infection) [18]. Studies have additionally measured enforcement strategies (e.g., police crackdowns, arrest, condom confiscation) which can similarly impact women’s safety and harm reduction around STI and HIV transmission and infection [19]. For instance, frequent arrest, crackdowns, and move along-tactics that displace FSW to unfamiliar areas can increase vulnerability to violence and move women further away from outreach and HIV prevention services [20] and are associated with client violence [21]. Condom confiscation can prompt unprotected sex and has been associated with STI infection [22, 23]. A large proportion of street-based FSW in settings including the U.S. are also illicit drug users and regularly face the dual risk of negative police interactions and arrest associated with drug use [24–26]. Studies have shown the adverse impacts of drug-related enforcement practices on HIV, including a positive association between syringe confiscation and HIV infection among injection drug users [27, 28]. Law enforcement practices such as enhanced police surveillance represent a major impediment to utilization of syringe services programs and other harm reduction approaches [29, 30] that represent one of the most effective ways to protect rights and limit the human suffering associated with addiction.

In response to this accumulating evidence, attempts to forge partnerships between sex worker organizations and the police across diverse settings (including India, Nepal, Thailand, Australia) have been documented. Interventions have included improving communication between the local sex worker community and law enforcers, police trainings, and introduction of new policies to improve sex worker safety [31]. In the U.S. there is a dearth of intervention work directly addressing the policing of sex workers. However, in the broader context of the failed ‘war on drugs’ [32] - a campaign in the United States since the 1970s to combat illegal drug use by large increases in enforcement, penalties, and incarceration for drug offenders - the U.S. is now mainstreaming public health-oriented harm reduction interventions aimed at illicit drug use [27, 33–35] which has implications for many FSW who also use drugs. Studies have evaluated the effectiveness of police trainings and policy changes (e.g., diversion programmes) aimed at shifting police attitudes and practices, while securing police buy-in through a lens of occupational health and reducing rates of recidivism [36, 37, 71].

Despite some examples of positive steps to address policing’s contribution to the HIV risk environment of two overlapping vulnerable populations, evidence suggests that shifting police culture and gearing day to day patrol practices towards public health orientated goals is both complex and challenging. In Krüsi et al. [1], a qualitative exploration of FSWs’ policing experiences under a new Canadian policing policy intended to prioritize sex worker safety, the authors found that stigmatizing attitudes overshadowed and undermined any improvements to police protection. More studies are needed that focus on understanding the mechanisms and drivers that underlie police behavior and decision making. Such insights are critical to the design of interventions and police public health partnerships that address the health and rights of FSW and other marginalized populations. Current research is heavily based on quantitative epidemiological surveys and/or self-report qualitative interviews with FSW, but not police. Burris [38] has suggested that research has missed the opportunity to focus on looking at the behavior of the police towards vulnerable populations and the drivers for it, thereby creating avenues from which to design interventions that address the underlying causes of the negative health and human rights impacts of policing.

Ethnography engenders analysis that attends to the variance in and contextual specificity of policing taken towards marginalized groups. This approach allows for an examination of the gap between ‘laws on the books’ [38] (i.e., criminalization of sex work that demands arrest) and the realities of patrol officers’ day to day practices. Bittner’s [8] formative work on Skid Row and many subsequent ethnographic studies have emphasized the discretion and autonomy with which street-level officers deploy the law and legal enforcement policies, with law often providing the legitimacy and authority to impose a variety of unchecked practices [8, 39]. To understand what shapes police behavior towards vulnerable groups, the concept of police culture, defined as the shared norms, beliefs and values that shape police behavior [40], is important. Traditional accounts of police culture suggest a homogenous police mentality whose values can be highly problematic with respect to vulnerable groups and human rights compliant policing. Cultural racism, for instance, has manifested in over-policing and abusive practices towards black and other minority groups [41, 42]. In the field of domestic violence policing, elements of traditional police culture and a masculine ethos that frames ‘real policing’ as the policing of violent crime or large drug seizures, has been shown to influence officers’ exercise of discretion and dismissive attitudes to domestic violence work [43].

 More contemporary accounts of police culture have moved away from a ‘monolithic’ interpretation of police culture, instead exploring the influence of broader social and political conditions, organizational considerations (e.g., leadership, supervisory styles) and individual officer occupational styles and agency in shaping behavior [42]. Neighborhood context has also been shown to influence police behavior. Klinger in his theory of policing explores the connection between police decision making and their ecological environments, putting forward the proposition that officers police less in high crime areas (e.g., viewing victims as less deserving, and crime as more normalized [44]. Work by Chan [45] exemplifies further efforts to understand the impact of the broader ‘policing environment’ in shaping officers’ working culture.

This study seeks to identify some of the underlying drivers shaping police practices that have been previously examined in terms of their impact on FSWs’ health and HIV risk. In particular, the present study aims to explore what factors influence: *a)* police practices already identified in the literature as adversely affecting street-based FSWs’ HIV risk and human rights; and *b)* police officers’ ability and willingness to adopt a more public human rights orientated approach to street-based sex work. Through the observation of patrol officers in Baltimore, a major U.S. city, we explore these factors, with particular attention to the broader ecological environment in which policing occurs. By ecological environment/context we are referring to economic, social and political factors, in addition to broader physical environmental influences [38]. ‘Street-based’ sex workers refer to those who almost exclusively solicit clients on the street or in public places (e.g., parks), and are the focus of this study. Although street-based FSW represent a small section of those working in the broader sex industry, they are also the most visible and therefore vulnerable to policing tactics and abuse [46], as well as most urgently in need of improved police protection from violence [12]. Our methods were designed to produce a nuanced description of the policing of street-based sex work to better inform future health and human rights and public safety interventions that mitigate the established negative health impacts of policing on street-based sex workers.

### Context and research setting

Baltimore is an East Coast city that has been challenged by a sustained history of social and economic hardship, largely divided along racial lines, with white prospering neighborhoods sitting in stark contrast to impoverished and largely African American ones. The 2018 American Community Survey reports that 21.8% of city residents live below the poverty line [47]. Deeply embedded structural inequities are rooted in the decline of the city’s manufacturing industry which led to major demographic change within the city, often referred to as ‘white flight’, but also marked by the later exodus of Baltimore’s black middle class [48]. Today Baltimore's low density city is best described as enclaves of wealth nested within areas characterized by extreme poverty. Street-based sex work, all aspects of which are criminalized (buying and selling) in the state of Maryland, is predominantly located in spaces that are within or on the borders of Baltimore’s surviving working class communities and the most hollowed-out neighborhoods. The latter neighborhoods often have the highest density of open-air drug markets and violent crime, with vacants outnumbering inhabited homes. Street-based FSW in this setting are characterized by high rates of STI infection and co-morbidities related to drug use and violence [21, 49, 50]. In particular, studies in this setting highlight the magnitude of the overlap between street based sex work and injection drug use. Amongst a cohort of 250 cis-FSW recruited in Baltimore City, 70% reported daily heroin use and 60% reported ever having drugs or drug paraphernalia confiscated [21]. Despite the city’s predominant black population, a number of the main sex worker strolls in Baltimore are dominated by white drug-using women, a group who in many ways embody the city’s growing urban poverty.

Dating back to the early 1990s, the police response to sex work and illicit drug use was one of ‘zero tolerance’, characterized by large number of stops, searches, and arrests [51]. During the time that this study was conducted, Baltimore city saw a surge in homicides, with 344 murders recorded for the year of 2015 when data collection commenced (up from 211 murders in 2014) and a police department under unprecedented scrutiny and pressure [52]. This scrutiny was occasioned by the death of Freddie Grey, a 25-year old black African American whilst in police custody. A subsequent report by the US Department of Justice concluded that Baltimore Police Department (BPD) requires widespread reform. Findings included unconstitutional stops and searches, and excessive use of force by officers, further eroding already fragile community trust in law enforcement. Although sex workers were not considered as a distinct population by the Department of Justice investigations, the report suggested that there could be a gender bias affecting police treatment of vulnerable women in the reporting of sexual assault, including FSW [73]. Our ride-alongs occurred in police districts characterized by these abuses and police deficiencies, as well as the socioeconomic disparities that foster the police department’s ongoing major enforcement challenges.

## Methods

Ethnographic methods (i.e., key informant interviews, observational ride-alongs with police officers during their shifts) were employed in four police districts in Baltimore. In selecting these police districts, we first looked at open-access Baltimore arrest data between 2013 and 2015 for prostitution and drug arrests, selecting arrest records for females only (Open Source Baltimore). Districts that had higher levels of prostitution or prostitution and drug arrest activity were selected. Secondly, we held meetings with the Commanders at identified districts and leadership at outreach organizations (e.g., Baltimore Needle Exchange Program) who validated our initial choice of districts based off the arrest data. This paper reports on observational fieldwork conducted in these districts between 2015 and 2016.

The research team consisted of six field observers all trained in ethnographic observation techniques including social epidemiologists (KF, SP, SL), a criminologist (BS), an anthropologist (VK) and a research assistant with extensive knowledge of Baltimore City and our target population (SH). Observational ride-alongs with officers were conducted throughout the year during a variety of shift times to maximize variation in observations and officers. In addition to routine patrol officers, ride-alongs were done with two other specialized units that focused on sex work and drug policing. A total of 54 ride-alongs (281 hrs) were completed between July 16th 2015 and February 25th 2016 with 64 unique officers. The majority of ride-alongs (*n* = 46) were with routine patrol, and a small number were with two specialized units, described below, *n* = 2 with Vice and *n* = 6 with District Operations.

Vice is a centralized unit that conducts undercover stings to arrest sex workers and clients, and conducts enforcement around sex trafficking. In addition, district-level operations units consist of plainclothes officers whose role includes conducting undercover drug purchases and street stop investigations to build cases against violent offenders and drug organizations. All officers provided verbal informed consent and field observers accompanied them for a 2–10 h period of a 10-h shift, with an average ride-along lasting 5 h. A field note aid and an encounter form were used to help field observers structure note taking (e.g., features of environment, policing priorities, officers’ attitudes/views, interactions, routines and practices), capture specific elements of police-FSW encounters (e.g., location, nature of incident, actions, use of discretion), and ensure consistency across observations. At the same time fieldworkers were also encouraged to record observations, reflections, and even idiosyncratic details that enabled them to better contextualize their experience as it occurred in the moment, while preventing misinterpretations that might occur if quotes or observed details were decontextualized. Observers generally rode along with a single officer and sat in the front of the vehicle, which aided conversation and rapport building. During street stops and citizen encounters observers would, if safe and appropriate, leave the patrol vehicle and observe exchanges. Field-notes were written up within 48 h by observers to minimize recall bias. Stand-alone quotes from field notes or quotes integrated within a field note are italicized to denote they are the direct quotes. All identifying information has been removed to ensure anonymity of the police districts and those observed. Field notes and interview extracts are labeled with the ride-along officer(s) type or rank, gender, race and age.

Key informant interviews (*n* = 10) were conducted with police leadership (e.g., District Commanders, Community Liasons, Vice). Sampling was purposive, based on knowledge of the research team and opportunistic, with interviews limited to those available and willing. All key informants provided verbal informed consent and were interviewed in a quiet private location, usually their office. Seven key informant interviews were audio recorded. Extensive notes were taken during the 3 remaining interviews. Notes were written up within 48 h of an interview and audios transcribed verbatim. Key informant interviews explored organizational attitudes among senior officers around policing sex work, attitudes towards public health-orientated approaches to policing marginalized populations, opportunities and challenges to improve relationships, and alternative policing approaches towards the FSW community in Baltimore City. All key informant quotes are anonymized except for the type of officer interviewed (e.g., District Commander, Vice).

Ethnographic data was analyzed adapting a pluralistic theory and data driven approach, that was both inductive and deductive [53]. Initial open-coding involved reading and line-by-line analysis of typed field notes from all 4 districts and key informant interviews by the first author to categorize and conceptualize the data by hand. This led to an initial codebook that was applied by the coding team using Nvivo software to an additional number of field notes and then revised iteratively by the coding team to improve the scope of the final codes and sub-codes. Team feedback on emergent categories built an audit trail to ensure the inclusion of multiple perspectives and enhance reliability [54]. Once coding was complete the first author moved to concept level categories, informed by the concept of police culture [42], including how broader ecological factors can shape police culture [44], as well as complementary criminological and sociological literature exploring the more explicitly spatial exercise of police discretion [55, 56].

Approval for the study was obtained from the ethics committee at Johns Hopkins Bloomberg School of Public Health and conducted in accordance with a pre-obtained memorandum of understanding entered with Johns Hopkins Bloomberg School of Public Health and the Baltimore Police Department.

## Results

Of the 64 officers in our study, 86% identified as male and 14% identified as female. Approximately 63% of ride-along participants were white and 27% were black, the remaining were Asian or other. Thirteen percent of participating officers identified as Hispanic. Officers’ years of experience ranged from less than one year up to 34 years, with the average being seven years. Most officers reported that they did not live within Baltimore City.

Our observations with police in Baltimore City and key informant interviews with police leadership identified important themes that: 1) influence ongoing enforcement practices known to have a detrimental impact on FSWs’ health and rights; 2) impact non-enforcement that have implications for FSWs’ health and safety; 3) shape police’s continuing failure to protect FSW, including addressing sexual violence and 4) impede a shift towards a more public health and human rights orientated approach to policing sex work. Our results point to ecological factors at the structural, organizational, and community levels that shape individual level police practices and underpin police attitudes. Based on our findings, the discussion addresses the implications of this study for intervention and policy development to drive forward a public health and human rights centric policing of sex work.

### Factors influencing ‘police enforcement’ practices towards FSW

Observations in this study highlight key ecological factors driving police enforcement approaches. Patrol officers, whilst rarely able to arrest sex workers for prostitution (due to the high evidential requirements), are under intense pressure from community associations and residents to actively police sex work, particularly where it becomes concentrated in gentrifying neighborhoods. As this observation note illustrates, both the physical and temporal geography of the area has a significant impact on policing practices:

The officer explains that he likes to keep them [FSW] north of [street omitted]. If they go too far south of there he gets a lot of complaints. He doesn’t really get any complaints if they’re kept in the *“seedier areas.”* The same is somewhat true for times, he explains he gets really concerned if it’s 7 AM and the kids are going to school with the sex workers on the street, where as 4 AM is not so much of a problem. (Patrol Officer, Male, White, 26).

The majority of officers’ approach in these residential spaces focuses on ‘move along’ tactics aimed at displacing women back to areas where communities are less likely to complain. Instead of arrest, other tools of law enforcement (e.g., contact sheets, open warrant checks, parking and watching FSW) are utilized to lessen sex work visibility. No patrol officers or command seemed to be aware of the public health and safety implications of move along tactics on FSWs’ health and safety, but most agreed that displacement of FSW was not an effective solution to policing sex work. Rather it appears to be a police response to prevailing and persistent community intolerance of FSW in more gentrified communities:

The officer explained that he received a lot of community complaints from the wealthier ‘white’ residents, both sex worker related and non-sex worker related, *“They might as well call you a neighborhood watch.”* (Patrol Officer, Male, Black, 32).

Consistent with other literature [56, 57], our findings also pointed to community association and homeowner complaints being a strong driving force behind FSW-targeted Vice ‘sweeps.’ Vice, unlike patrol, are responsible for conducting ‘stings,’ during which plainclothes officers solicit services from women to provide the necessary grounds for a prostitution arrest. However, an additional ecological factor driving these ‘sweeps,’ alongside community pressure, is the periodic need to respond to upticks in violent crime, such as shootings.

*“There has been a crime initiative the last two weeks as the crime is out of control, so we put boots on the ground including Vice. The last two weeks we’ve just been out rounding the women up* [FSW]*.”* (Vice Command).

While FSW themselves do not commit these violent crimes, they often observe crimes occurring on the street and can provide the police with valuable information. As this Vice officer explained during an observation of a sting operation:

I took the opportunity to ask the officers whether they thought what they were doing was getting women (FSW) off the streets. The Vice officer responded as he let out a laugh: *“Ha. Not at all. They’ll be walking the streets again as soon as they get out (of jail). We’re out here for information basically.”* (Vice Officer).

### Factors influencing ‘non-enforcement’ approaches to sex work

Observations revealed that in marginalized and high-crime city locations, patrol officers do not police sex work as aggressively as they do in more residential and gentrified areas. Marginalized neighborhoods are characterized by few residential complaints and high levels of drug activity and violence. Observers’ field notes consistently documented that most police take no action to displace FSW in these areas, despite frequent sightings of sex workers actively working the strolls. Additional factors shape patrol officers’ approach to enforcing or not enforcing drug laws. A neighborhood context in which illicit drug use, in particular heroin, is ubiquitous and police prioritize calls for service associated with violent crime, is coupled with variations in individual officers’ outlooks. Whilst ‘rookies’ seem more enthusiastic about making low-level drug arrests, more seasoned officers seem more cognizant of the bureaucracy entailed in arresting vulnerable groups such as FSW, and of the limitations on their time. As this patrol officer explained:

*“She* [FSW] *probably had a warrant if I had run it.”* However, he doesn’t want to find out because *“if she goes to central booking, she won’t pass medical and then I have to take her to the hospital and stay until she’s cleared before taking her back to booking.”* He says they’re already short on officers, *“We try to avoid locking up certain groups … handicapped people,”* or other people who might not pass their medical. “*Once you have a couple years on you, you know these things,”* he says. (Patrol Officer, Male, Asian, 32).

However, the mere potential to invoke drug related arrests, remakes FSW as important assets for intelligence. District commanders described FSW as *“the Google search engine of the street.”* Although some FSW are paid informants, more commonly the exchange of information is informal and drives the frequency of interactions in marginalized spaces between police patrol and FSW. These exchanges are predominantly characterized as simply ‘checking in’ and being friendly to FSW, although as this quote illustrates there is almost always the underlying coercive threat of enforcement:

The officer started talking about FSW as informants, *“We will buy them food all the time, or now with it getting colder, we’ll get them hot chocolate or coffee. They will literally tell you everything.*” Another officer chimes in, *“The dealers and stuff don’t treat them good, so they have no problem giving up information on them.*” He elaborated, *“We have a good relationship with them. We leave them alone* [i.e. don’t arrest]*, and in return they give us information.”* (District Operations, Male, White, 24).

It appeared that these exchanges primarily contribute to many officers’ intimate knowledge of FSWs’ lives and vulnerabilities (e.g. homelessness, lack of employment opportunities, hunger, need for wound care). However, we rarely witnessed officers utilize this knowledge and daily contact with FSW to offer any bridge to health or social services. Instead, observations highlighted that where help is given it is often ad hoc and predominantly reflects a complexity of moralistic, gendered and paternalistic attitudes by individual officers, with no guiding organizational norms around public health policing:

*“Sometimes I do run into women who are genuinely selling their pussy to make ends meet – feed their kids, pay rent, etc., and when that happens I try to connect them with some services.”* (Patrol Officer, Female, Black, 35).

*“I try to help them, I really want to help them. I’ll give them my cell and tell them to call me on a specific date, if they call me on that date then I know they are serious and will do what I can to get them help.”* I ask the officer how many have called on the date, *“One,”* he replies. (Patrol Officer, Male, White, 37).

### Factors hindering police protection and assistance to FSW

The majority of officers appeared to view violence towards FSW as an inescapable part of the street existence, as opposed to crimes against vulnerable women that properly deserve police attention.

The first thing the female officer brought up was how they [FSW] are routinely victimized while engaging in sex work, such as being robbed or assaulted by clients. She then stated, *“After they get messed with, they call us and report it! Can you believe that?! And we have to deal with it. Could you imagine if a drug dealer reported to us that someone beat him up while selling or took all his drugs? It’s unbelievable!”* (Patrol Officer, Female, Black, 35).

Officers’ attitudes towards FSW as less deserving of police assistance and protection emerged as closely linked to broader stigmatizing attitudes to FSW in this context, shaped by both their identity as sex workers and drug users. This was underscored by the frequent documentation during ride outs of dehumanizing police banter including phrases such as *“pregnant prostitute junkie”, “skanky”, “disgusting”* and descriptions of FSW drug related sores and poor physical condition. One officer referred to a FSW he knew on the strolls simply as *“abscess”.* Some of the most dehumanizing language involved the use of animal imagery, including describing FSW as junkies waking up for *“feedin*g.”

A small number of officers did describe FSW safety as more of a priority and provided examples of proactively addressing FSWs’ needs. This emerged at the level of the individual officer and did not appear to translate into a more generalized organizational concern to address and prioritize FSW safety. Although, during key informant interviews police commanders would give anecdotal examples of their officers assisting women by buying food, bringing them warm clothes, and taking them to local service providers.

During the time that ride-alongs were conducted, a significant institutional shift towards the broadening of collaborations between public health and public safety was instigated by the BPD. This involves officers carrying Naloxolone (narcan) for overdose victims. However, as this vignette illustrates, patrol officers’ attitudes often embodied a lack of sensitivity and embedded ‘canteen (discriminatory) culture’ [72] towards FSW who inject drugs:

She [FSW] had nodded out from heroin and was laying on the steps but was still breathing. *“Wake up, ma’am! Ma’am! Ma’am wake up!”* the officer said to her, with no response. He then shouted, *“Narcan!”* and was about to use it but she shot up to her feet instantly and began walking away. She made it about a block and a half away when she hunched over and fell asleep standing up. He again yelled, *“Narcan!”* and she woke right back up and walked away. *“Man! This Narcan stuff is great! It works so good you don’t even have to use it!”* he said. Everyone shared a laugh and the female officer showed us a video entitled ‘Baltimore Gold’ of a women nodding out from heroin in a parking lot and a male that was with her trying to keep her awake. (Patrol Officer, Male, White, 29).

### Identifying barriers to more public health and human rights orientated policing of FSW

In interviews with district commanders the phrase *“you can’t arrest your way out of the problem,”* was frequently quoted with respect to FSW. However, in talking about the alternatives, there was recognition at an organizational level of the complexity and scope of FSWs’ vulnerabilities and the considerable gap between understanding traditional law enforcement isn’t the solution, and viable public human rights orientated policing approach.

The Captain reflected, *“How do you stem the tide? When you get one off the street, get them in a re-entry type of program to provide services, what do you do with the next one? How many officers do you need to deal with all the women on* [stroll name omitted]*? We need long term goals.*” (Key Informant, Major).

Command also recognized the realities of day-to-day patrol policing and organizational resource constraints (e.g., shortages of officers, other policing priorities, lack of specific training) considerably hamper a proactive response. This was backed up by observations with patrol where field observers noted understaffed shifts, backed up calls for service, and a focus on the surge in violent crime. Interviews with District Command advocated for organizational level improvements to officer trainings, aimed at better equipping officers for the *“social worker aspects of policing”* (District Commander, Male, White, 45). However, most patrol officers sense of low self-efficacy around helping FSW focused on the more structurally embedded lack of external support from other agencies, coupled with a fatalistic attitude towards FSWs’ prospects of finding a way out.

*“People expect too many things from us – we’re not social workers.”* He says, *“I feel sorry for them, same as for young guys on the corner selling drugs. But it’s not our primary duty to deal with them directly. There are other agencies getting paid* [to provide these sort of services]*, but where are they? It takes an act of God, damn near, to get them to help them* [the women] *– they are failing all of us.”* (Patrol Officer, Male, White, 35).

The officer explains that in his experience there’s not really much you can do - sex workers are stuck in what he calls a *“revolving door.”* (Patrol Officer, Male, White, 47).

Crucially, the broader structural context of criminalization and stigmatization emerged as hampering a shift in policing at an individual officer and organizational level towards public health and human rights approaches. Despite many patrol officers’ negative attitudes to FSW, many advocated that a first step would be to move away from criminalization:

*“Don’t tie my name to this but they just need to decriminalize it. From talking to these girls out here you see they have so many issues and they see drugs and prostitution as their only way out. If it was decriminalized, and I don’t know what kind of [employment] benefits they would have or what that would look like, maybe you can figure that out, but then they could get the help they need.”* (District Operations, Male, White, 24).

The same sentiment was reflected by almost all senior police leadership at the time of the study. Although Vice officers did not specifically mention decriminalization, Vice command indicated that the units resources and core work had shifted to investigation of human sex trafficking.

## Discussion

Our ethnography provides some of the first evidence generated from studying police that both elucidates and confirms factors shaping police practices towards FSW, largely absent from the public health and social science literature to date. The study seeks to complement and bridge the divide in the existing literature, which on the one hand has included prominent policing ethnographies [7, 9, 58] investigating police practices towards other vulnerable populations, and on the other socio-epidemiological literature specifically focused on documenting police’s contribution to FSWs’ risk environment [13, 16]. In seeking to identify the underlying factors that influence the policing of FSW, our findings provide a platform from which to discuss how to most effectively intervene to alleviate the negative health and human rights impacts surrounding the policing of FSW in both the U.S. and other contexts.

Social-spatial control through law enforcement tactics has been highlighted by criminologists and sociologists to aid the ‘cleaning up’ of urban spaces [44, 59, 60]. In this study, “move along” tactics by patrol and ‘sweeps’ by Vice, were shaped by an ecological context in which more affluent neighborhoods are dominated by communities’ moral concerns (e.g., children seeing sexual activity on the way to school, discarded condoms on porches). Interestingly, many patrol officers expressed frustration toward community complaints, which they viewed as born of social anxieties rather than actual crimes, and disproportionately drawing on already overextended patrol resources. These findings confirm existing literature [1, 56] that point to the importance of gentrification and community level stigma in driving an ultimately ineffective police response to sex work that compromises FSWs’ safety. An interesting additional contextual factor shaping displacement of FSW by the police, are the periodic upticks in violent crime that characterize cities such as Baltimore. Spikes in violent crime prompt crackdowns by Vice; these sweeps are not intended to address sex work, but utilize the threat of arrest to coerce FSW into providing criminal intelligence. These tactics displace FSW from their usual strolls as they attempt to avoid such crackdowns, and highlight the coercive nature of police relations with FSW that can contribute to a climate of vulnerability in which FSW rarely view police as interested in their rights as individuals or collective wellbeing. Public health research additionally highlights the direct and indirect impact these different spatial displacement tactics can have on FSWs’ safety and HIV risk factors (e.g., client violence, condom use, current STIs) [13].

Our results indicate that neither patrol nor command view legal enforcement tactics as effectively addressing sex work. Instead officers are engaged in reproducing socially dominant ideas from local communities about what is criminal conduct, regardless of actual evidentiary standards for those crimes and despite its potential to undermine FSW safety. Interventions need to focus on educating police departments and the communities they serve about the public health implications of these policing tactics. Through public safety and health partnerships and officer training, police departments could work in these communities to highlight the ineffectiveness of existing policing approaches and question communities’ categorization of FSW as a public nuisance, rather than a public health issue. Such steps could do much to alleviate the presently damaging spatial control of FSW in this study context, and others. Additionally, in the Baltimore context thought needs to be given to incentivizing police officers to live within the city (at present the majority do not) as a way of gaining community consent and legitimacy. It is potentially harder to address organizational police policies that utilize prostitution enforcement as a tool for gathering criminal intelligence, where there is such obvious police capital. Instead this example of police misuse of power serves to support and emphasize the importance of decriminalization in dismantling the legal apparatus under which police discretion is used to exploit sex workers in ways that not only undermine their health and human rights, but limit any trust that could be fostered between this population and the police.

In line with other policing studies, enforcement practices in ‘marginalized spaces’ such as open-air drug markets contrasted enforcement in ‘prime spaces’ such as more affluent residential neighborhoods discussed above. As Stuart [61] explains, ‘police discretion is, first and foremost, contingent upon how officers conceive the neighborhoods they patrol’. Within the milieu of the open-air drug trade, FSW were not prioritized as targets of sex work-related patrol enforcement at all, instead it was their identity as injection drug users that shaped patrol interactions. Observations revealed considerable variation in drug enforcement based on patrol officers’ discretion. Less experienced patrol officers were characterized as being more willing to conduct drug arrests, while more experienced officers recognized the bureaucracy entailed in low level arrests and the lack of real police capital. In contrast, all officers utilized the underlying threat of drug arrests to extract information on violent crime. Many police officers tended to achieve this by building relationships with FSW, developing considerable knowledge of women’s vulnerabilities along the way. Interventions that seek to shift police’s role towards helping women (e.g., on the spot referrals for HIV testing and counselling), could consider how such frequent day-to-day interactions with FSW and knowledge can be utilized to bolster FSW right to access social and health related services. This would require a shift away from what Blankenship et al. characterized as the ‘*diffusion of criminalization’* [62], whereby FSW and people who use drugs have their identities defined and limited to that of a criminal, as opposed to women working in a risky environment. Although during observations police interactions with FSW often appeared friendly and good-natured, they are underscored by unequal power dynamics and continuously shaped by the broader structural forces of criminalization of sex work and illicit drug use.

Consistent with findings in other settings [63, 64] police confirmed high levels of violence experienced by FSW, which has been linked in the public health and human rights literature to FSWs’ risk of HIV acquisition. In addition, studies - most recently in the U.S. - have found that FSW who have experienced abusive police practices are more likely to experience client violence [21]. Officers acceptance of FSWs’ violence as inevitable was reinforced by the violent context in which FSWs’ lives are situated, and appeared to act as a normalizing factor underscoring officer attitudes. In addition, it is likely that officer’s tendency to rank FSWs’ experience of sexual violence as a low policing priority compared to other violent crime is also rooted in larger gendered patterning of police culture, including victim blaming and patriarchal attitudes towards women [65, 66]. Consistent with broader criminology literature [67] female officers observed in this study appeared to project a hegemonic masculine police culture. This was reflected in the way most female officers, as well as male, dismissed FSW as deserving victims in cases of client rape and the gendered language they used to describe women during ride outs. Additional research is needed amongst FSW populations to unpack the complexity and layering of the relationship of female officers to FSW as victims of crime. This should include understanding the silencing of some forms of violence against FSW based not only on gender but on race, social class and other stigmatized identities such as sex work and illicit drug use [66].

Only a small number of officers’ patrol interactions with FSW reflected a more guardian (i.e., protective policing) style. Furthermore, observations in this setting revealed little evidence of police proactively helping women (e.g., with social service, drug program referrals), coupled with evidence of deeply embedded stigmatizing attitudes. Studies have highlighted the dehumanization of vulnerable populations by the police, often connected to the spaces they occupy [7, 9, 39]. In this study, both male and female officers’ language (e.g., ‘pregnant prostitute junky’) reflected intersecting stigmatizing normative attitudes toward FSWs’ sex work and injection drug use as spoiled identities and manifested in police officers attitudes of FSW as undeserving victims. There was an extraordinary tension between many officers’ intimate knowledge of FSW lives and moments of empathy, and their frequent dialogue of ‘othering’. Legal literature has highlighted the role of legal controls in reinforcing negative stereotypes and perpetuating social exclusion and vulnerability [68]. Similarly, Blankenship et al. in their qualitative study exploring injection drug using FSW experiences with police in Colorado, USA found that women’s “… identity is reduced by both law enforcement and the public health system to a single act that is illegal … That many individuals have families is forgotten.” [62]. Here observations suggested that the relentless exhaustion and sense of futility embodied in police attitudes and practices are amplified by a structural environment of poverty, criminalization of sex work and substance use that reinforces officers’ stigmatizing attitudes.

Collectively our findings highlight the need for more far reaching structural changes to address the broader environmental context in which the policing of sex work operates. In particular, patrol officers cited the need to remove criminalization of sex work and ensure there is adequate and integrated external support from health and social services as key to better human rights orientated public health and safety partnering. Police command also pointed to the very real organizational limitations to prioritizing public health partnering around vulnerable populations, such as sex workers. Limitations include a lack of dedicated financial resources and considerable constraints on police time, with drastically understaffed shifts being the norm. It is these bigger shifts in the structural landscape that will ultimately pave the way to a new era of public health informed policing towards sex work, and could ensure similar systemic changes to those that occurred around the policing of mental health [69]. Additionally, further unpacking and addressing the complexity of intersecting stigmatizing assumptions of police officers towards women in this setting is critical. Police sensitivity trainings conducted in other settings (Erausquin, Reed, and Blankenship 2015), including peer education of officers in collaboration with sex worker organizations (Tenni, Carpenter, and Thomson 2015) could be explored in this context. In particular, such collaborations need to address the complex overlap between stigmatization of sex work, illicit drug use, poverty and the situational ‘othering’ of FSW, for whom normal social entitlements do not apply [70]. Trainings however are insufficient without an organizational shift towards prioritizing FSWs’ human rights and health, in particular, introducing policies and systems of accountability that require police to respond equitably to FSWs’ experiences of violent crime. Another important dimension of officers’ embedded stigmatization towards FSW surrounds their injection drug use. In line with ongoing political and policy shifts at a national and state level to introduce pre-booking diversion for low level offences in the U.S., Baltimore City has recently joined the ‘LEAD’ program [71]. LEAD introduces diversion to drug and social services at the point of arrest, albeit currently targeted specifically at drug using populations, rather than FSW specifically. Despite this, it presents an opportunity to better institutionalize harm reduction and public health thinking into policing practices around vulnerable populations' drug use. It also has the potential to ensure a more uniform approach to police officers current highly discretionary interactions with low level drug users, including FSW.

The study was characterized by several limitations. In setting out to conduct this ethnography with the BPD, the scrutiny around police practices may have led police to be less frank in their opinions. The purposive and opportunistic nature of our observational sampling meant that there may have been the opportunity for districts to pick ‘by the book’ officers for field observers to ride-along with, meaning we missed the perspectives of more frank officers. The nature of observations also meant that there were no expectations around witnessing the most egregious police behaviors towards FSW during ride-alongs, despite these having been documented in work with FSW in this setting [21]. Despite these limitations, the study was able to establish an open and frank dialogue with the police department around the aims of the study through initial key informant interviews, allowing us to capture an honest portrayal of policing approaches and priorities towards FSW. Although the presence of observers in the field can influence officers’ actions, field notes revealed the extent to which officers let down their guard and shared frank opinions during observations. In turn it is acknowledged that the ethnographers involved in this study carry with them their own sources of bias, particularly given their work with stigmatized populations like sex worker populations. Diversity in background and experience (including gender, time in the city, previous work with the police) was intended to help mitigate the effect. A further limitation of the data relates to the lack of focus on race as a socio-structural category that may have had a bearing on our understanding police-sex worker interactions. FSW that were observed and encountered during ride-alongs tended to be white. It is beyond the scope of this paper to unpack the reasons for encountering a predominantly white street-based FSW population in the context of city where economically deprived neighborhoods are disproportionately black. The predominance of white FSW could be tied to the relationship between street-based sex work and the drug economy, or a preference among other FSW populations to utilize indoor venues.

## Conclusions

In conclusion, much can already be garnered from current public health and human rights literature on the types of police practices that compromise FSWs’ health and safety, particularly as it relates to HIV and violence. Still, a more in-depth understanding from the policing side of the circumstances that drive law enforcement approaches to street-based sex work has been lacking. Such an understanding is critical to the collaborative design of interventions with police in different settings. In considering public health-police partnerships to address the needs of FSW populations in the U.S. and elsewhere, this study supports existing calls for decriminalization of sex work, supported by institutional and policy reforms (e.g., around coercive police practices, FSW street safety), neighborhood-level dialogues that shift the cultural landscape around sex work within both the police and larger community and innovative individual-level police trainings (e.g., to address sex work and illicit drug related stigma). Points of leverage include the general acceptance of officers around the need for change and a frustration with ineffective policing strategies towards sex work. A shift in police culture that questions communities’ moral sensibilities around sex work and categorization of FSW as a public nuisance, rather than a vulnerable group in need of health and social support could help alleviate the damaging spatial control of FSW that have been shown to promote HIV risk behaviors (e.g., rushing negotiations, screening clients, and condom use). Many officers of all ranks favored some form of legalization or decriminalization, which is a view that is heavily supported by the public health literature that shows both the enormous negative health implications of criminalization for sex workers and the impact that decriminalization could have on the course of the HIV epidemic among sex worker populations [13, 16]. This study highlights police support for this type of legal change in the State of Maryland, and suggests common ground on which police and sex worker activists could unify to instigate change. The unpredictability and discretion of policing practices towards FSW has the potential to significantly erode trust, finding ways to introduce some uniformity into FSWs’ interactions with police is key. One achievable policy reform that could promote trust and instigate a shift at the institutional level in the cultural landscape of policing sex work would be the introduction of a female sex worker liaison officer to facilitate follow up around FSW experiences of violence. However, police training for such a female police officer leadership role is crucial and would need to be rooted in an inclusive feminist perspective that moves away from the victim blaming of FSW.

Additionally, for such reforms to be effective, the study also highlights the importance of fostering extensive structural support for FSW and service referral options in collaboration with criminal justice reforms. Shifting police culture away from viewing the vulnerability of FSW as a resource to be exploited and towards viewing it as a harm to be reduced would require a seismic shift away from the current crime control climate, which is tightly bound to the city’s ongoing epidemic of violence and to the broader socio economic problems of Baltimore city. Finally, FSW must themselves be empowered in their legal and social rights, thereby holding police and civil society accountable for promoting health and equity amongst this population.

## Data Availability

Anonymized data may be made available on request subject to the Johns Hopkins Office of Human Subjects Research and the Institutional Review Boards and consistent with our funding body guidelines (NIDA) and memorandum of understanding with Baltimore Police Department.

## Abbreviations

FSW: Female sex worker
BPD: Baltimore Police Department

## References

[1] Krüsi A, Kerr T, Taylor C, Rhodes T, Shannon K (2016). ‘They Won’t change it Back in their heads that We’re Trash': the intersection of sex work-related stigma and evolving policing strategies. Sociol Health Ill.

[2] Goffman E. Stigma: Notes on the Management of Spoiled Identity. Englewood Cliffs: Prentice Hall; 1963.

[3] Link BG, Phelan JC (2001). Conceptualizing Stigma. Annu Rev Sociol.

[4] Benoit C, McCarthy B, Jansson M (2015). Stigma, sex work, and substance use: a comparative analysis. Soc Health Ill.

[5] Burke SE, Calabrese SK, Dovidio JF, Levina OS, Uusküla A, Niccolai LM, Abel-Ollo K, Heimer R (2015). A tale of two cities: stigma and health outcomes among people with HIV who inject drugs in St. Petersburg, Russia and Kohtla-Järve, Estonia. Soc Sci Med.

[6] Stuart F, Armenta A, Osborne M (2015). Legal control of marginal groups.

[7] Stuart F. Down, Out, and Under Arrest: Policing and Everyday Life on Skid Row. Chicago: Univ. Chicago Press; 2016.

[8] Bittner E (1967). The police on skid-row: a study of peace keeping. Am Sociol Rev.

[9] Goffman A (2015). On the run: fugitive life in an American City. Picador.

[10] Moskos P. Cop in the Hood: my year policing Baltimore’s Eastern District. Princeton: Princeton University Press; 2008.

[11] Brayne S (2014). Surveillance and system avoidance: criminal justice contact and institutional attachment. Am Sociol Rev.

[12] Sanders T, Campbell R (2007). Designing out vulnerability, building in respect: violence, safety and sex work policy. Br J Sociol.

[13] Platt L, Grenfell P, Meiksin R, Elmes J, Sherman SG, Sanders T, Mwangi P, Crago A-L (2018). Associations between sex work Laws and sex workers’ health: a systematic review and meta-analysis of quantitative and qualitative studies. PLoS Med.

[14] Shannon K, Goldenberg SM, Deering KN, Strathdee SA (2014). HIV infection among female sex Workers in Concentrated and High Prevalence Epidemics: why a structural determinants framework is needed. Curr Opin HIV AIDS.

[15] Rhodes T, Wagner K, Strathdee SA, Shannon K, Davidson P, Bourgois P, O’Campo P, Dunn JR (2012). Structural Violence and Structural Vulnerability Within the Risk Environment: Theoretical and Methodological Perspectives for a Social Epidemiology of HIV Risk Among Injection Drug Users and Sex Workers. Rethinking Social Epidemiology.

[16] Shannon K, Strathdee SA, Goldenberg SM, Duff P, Mwangi P, Rusakova M, Reza-Paul S (2015). Global epidemiology of HIV among female sex workers: influence of structural determinants. Lancet.

[17] Erausquin JT, Reed E, Blankenship KM (2015). Change over time in police interactions and HIV risk behavior among female sex Workers in Andhra Pradesh, India. AIDS Behav.

[18] Decker MR, Crago A-L, Chu SKH, Sherman SG, Seshu MS, Buthelezi K, Dhaliwal M, Beyrer C (2015). Human rights violations against sex workers: burden and effect on HIV. Lancet.

[19] Footer KH, Silberzahn BE, Tormohlen KN, Sherman SG (2016). Policing practices as a structural determinant for HIV among sex workers: a systematic review of empirical findings. J Int AIDS Soc.

[20] Shannon K, Kerr T, Strathdee SA, Shoveller J, Montaner JS, Tyndall MW (2009). Prevalence and structural correlates of gender based violence among a prospective cohort of female sex workers. BMJ.

[21] Footer KHA, Park JN, Allen ST, Decker MR, Silberzahn BE, Huettner S, Galai N, Sherman SG (2019). Police-related correlates of client-perpetrated violence among female sex Workers in Baltimore City, Maryland. Am J Public Health.

[22] Erausquin JT, Reed E, Blankenship KM (2011). Police-related experiences and HIV risk among female sex Workers in Andhra Pradesh, India. J Infect Dis.

[23] Wurth MH, Schleifer R, McLemore M, Todrys KW, Amon JJ (2013). Condoms as evidence of prostitution in the United States and the criminalization of sex work. J Int AIDS Soc.

[24] Beletsky L, Martinez G, Gaines T, Nguyen L, Lozada R, Rangel G, Vera A, McCauley HL, Sorensen A, Strathdee SA (2012). Mexico’s northern border conflict: collateral damage to health and human rights of vulnerable groups. Revista Panamericana de Salud Publica = Pan American Journal of Public Health.

[25] Odinokova V, Rusakova M, Urada LA, Silverman JG, Raj A (2014). Police sexual coercion and its association with risky sex work and substance use behaviors among female sex Workers in St. Petersburg and Orenburg, Russia. Int J Drug Policy.

[26] Werb D, Rowell G, Guyatt G, Kerr T, Montaner J, Wood E (2011). Effect of drug law enforcement on drug market violence: a systematic review. Int J Drug Policy.

[27] Flath N, Tobin K, King K, Lee A, Latkin C (2017). Enduring consequences from the war on drugs: how policing practices impact HIV risk among people who inject drugs in Baltimore City. Substance Use Misuse.

[28] Strathdee SA, Lozada R, Martinez G, Vera A, Rusch M, Nguyen L, Pollini RA, Uribe-Salas F, Beletsky L, Patterson TL (2011). Social and structural factors associated with HIV infection among female sex workers who inject drugs in the Mexico-US border region. PLoS One.

[29] Davis CS, Burris S, Kraut-Becher J, Lynch KG, Metzger D (2005). Effects of an intensive street-level police intervention on syringe exchange program use in Philadelphia, PA. Am J Public Health.

[30] Shannon K, Rusch M, Shoveller J, Alexson D, Gibson K, Tyndall MW, Maka Project Partnership (2008). Mapping violence and policing as an environmental-structural barrier to health service and syringe availability among substance-using women in street-level sex work. Int J Drug Policy.

[31] Tenni B, Carpenter J, Thomson N (2015). Arresting HIV: fostering partnerships between sex workers and police to reduce HIV risk and promote professionalization within policing institutions: a realist review. PLoS One.

[32] UNODC (2016). World Drug Report 2016.

[33] Beyrer C (2012). Afterword: police, policing, and HIV: new partnerships and paradigms. Harm Reduct J.

[34] Cusick L (2006). Widening the harm reduction agenda: from drug use to sex work. Int J Drug Policy.

[35] Jardine M, Crofts N, Monaghan G, Morrow M (2012). Harm reduction and law enforcement in Vietnam: influences on street policing. Harm Reduct J.

[36] Beletsky L, Agrawal A, Moreau B, Kumar P, Weiss-Laxer N, Heimer R (2011). Police training to align law enforcement and HIV prevention: preliminary evidence from the field. Am J Public Health.

[37] Silverman B, Davis CS, Graff J, Bhatti U, Santos M, Beletsky L (2012). Harmonizing disease prevention and police practice in the implementation of HIV prevention programs: up-stream strategies from Wilmington, Delaware. Harm Reduct J.

[38] Burris S, Blankenship KM, Donoghoe M (2004). Addressing the ‘risk environment’ for injection drug users: the mysterious case of the missing cop.

[39] Manning PK, Reisig MD, Kane RJ (2014). Ethnographies of policing. In The Oxford Handbook of Police and Policing.

[40] Loftus B (2010). Police occupational culture: classic themes, altered times. Polic Soc.

[41] Chan JBL. Changing police culture: policing in a multicultural society. Cambridge: Cambridge University Press; 1997.

[42] Loftus B. Police culture in a changing world. Oxford: Oxford University Press; 2009.

[43] Myhill A, Johnson K (2016). Police use of discretion in response to domestic violence. Criminology Criminal Justice.

[44] Klinger DA (1997). Negotiating order in patrol work: an ecological theory of police response to deviance. Criminology.

[45] Chan J (2004). Using Pierre Bourdieu’s framework for understanding police culture. Droit et Société.

[46] Krüsi A, Pacey K, Bird L, Taylor C, Chettiar J, Allan S, Bennett D, Montaner JS, Kerr T, Shannon K (2014). Criminalisation of clients: reproducing vulnerabilities for violence and poor health among street-based sex Workers in Canada-a Qualitative Study. BMJ Open.

[47] United States Census Bureau. 2018 American Community Survey 1-year estimates. Available at: https://www.census.gov/quickfacts/fact/table/baltimorecitymaryland/IPE120218#IPE120218. Accessed 23 Apr 2020.

[48] Levine MV. A third-World City in the first world: social exclusion, racial inequality, and sustainable development in Baltimore. Soc Sustainability Cities. 2000:123–56.

[49] Davey-Rothwell MA, Latkin CA (2008). An examination of perceived norms and exchanging sex for money or drugs among women injectors in Baltimore, MD, USA. Int J STD AIDS.

[50] Sherman SG, Park JN, Galai N, Allen ST, Huettner SS, Silberzahn BE, Decker MR, Poteat TC, Katherine H, Footer A. Drivers of HIV infection among Cisgender and transgender female sex worker populations in Baltimore City: results from the SAPPHIRE study. J Acquired Immune Deficiency Syndromes. 2019. 10.1097/QAI.0000000000001959. 10.1097/QAI.0000000000001959 30649029

[51] Collins R (2007). Strolling While Poor: How Broken-Windows Policing Created a New Crime in Baltimore. Geo J Poverty L Pol’y.

[52] Lowery W (2016). They Can’t kill us all: Ferguson, Baltimore, and a new era in America's racial justice movement. Hachette UK.

[53] Meyer S, Ward P. How to use social theory within and throughout qualitative research in healthcare contexts. Social Compass. 2014;8(5):525–39.

[54] Maxwell JA. Qualitative research design: An interactive approach. Thousand Oaks: Sage; 1996.

[55] Herbert SK. Policing Space: Territoriality and the Los Angeles Police Department. U of Minnesota Press. 1997.

[56] Hubbard P, Campbell R, O'Neill M, Pitcher J, Scoular J. Prostitution, gentrification and the limits of neighbourhood space. In: Atkinson R, Helms G, editors. Securing an Urban Renaissance. Bristol: Policy Press; 2007. p. 203–17.

[57] Lyons T, Krüsi A, Pierre L, Small W, Shannon K (2017). The impact of construction and gentrification on an OUTDOOR trans sex work environment: violence, displacement and policing. Sexualities.

[58] Weitzer R (2017). Recent ethnographies of the criminal justice system. Society.

[59] Beckett K, Herbert S (2008). Dealing with disorder: social control in the post-Industrial City. Theor Criminol.

[60] Yarwood R (2007). The geographies of policing. Prog Hum Geogr.

[61] Stuart F (2014). From ‘rabble management’ to ‘recovery management’: policing homelessness in marginal urban space. Urban Stud.

[62] Blankenship KM, Koester S (2002). Criminal law, policing policy, and HIV risk in female street sex workers and injection drug users. J Law Med Ethics.

[63] Deering KN, Amin A, Shoveller J, Nesbitt A, Garcia-Moreno C, Duff P, Argento E, Shannon K (2014). A systematic review of the correlates of violence against sex workers. Am J Public Health.

[64] Klambauer E. Policing roulette: sex workers’ perception of encounters with police officers in the indoor and Outdoor sector in England. Criminology & Criminal Justice: The International Journal of Policy and Practice. 1748895817709865. 2017.

[65] Radford J, Stanko E. Violence against Women and Children: the Contradictions of Crime Control under Patriarchy. In: Hester M, Kelly L, Radford J, editors. Women, Violence and Male Power. Buckingham: Open University Press; 1996. p. 65–80.

[66] Santos CM (2005). Women’s police stations: gender, violence, and justice in Sao Paulo.

[67] Rabe-Hemp CE (2009). POLICEwomen or PoliceWOMEN?: doing gender and police work. Fem Criminol.

[68] Wakefield S, Uggen C (2010). Incarceration and stratification. Annu Rev Sociol.

[69] Paterson C, Pollock E (2016). Editorial: global shifts in the policing of mental ill health. Policing.

[70] Vanwesenbeeck I (2001). Another decade of social scientific work on sex work: a review of research 1990–2000. Ann Rev Sex Res.

[71] Collins SE, Lonczak HS, Clifasefi SL (2017). Seattle’s law enforcement assisted diversion (LEAD): program effects on recidivism outcomes. Eval Program Plann.

[72] Hulst V, Merlijn (2013). Storytelling at the Police Station: the canteen culture revisited. The British Journal of Criminology.

[73] Department of Justice Report: Baltimore City Police Department (2016). U.S. Department of Justice Civil Rights Division.

